# An update of the WCRF/AICR systematic literature review on esophageal and gastric cancers and citrus fruits intake

**DOI:** 10.1007/s10552-016-0755-0

**Published:** 2016-05-06

**Authors:** Snieguole Vingeliene, Doris S. M. Chan, Dagfinn Aune, Ana R. Vieira, Elli Polemiti, Christophe Stevens, Leila Abar, Deborah Navarro Rosenblatt, Darren C. Greenwood, Teresa Norat

**Affiliations:** Department of Epidemiology and Biostatistics, Faculty of Medicine, School of Public Health, Imperial College London, St. Mary’s Campus, Norfolk Place, London, W2 1PG UK; Department of Public Health and General Practice, Faculty of Medicine, Norwegian University of Science and Technology, Trondheim, Norway; Division of Biostatistics, University of Leeds, Leeds, UK

**Keywords:** Esophageal cancer, Gastric cancer, Citrus fruits, Meta-analysis, Systematic literature review

## Abstract

**Purpose:**

The 2007 World Cancer Research Fund/American Institute for Cancer Research expert report concluded that foods containing vitamin C probably protect against esophageal cancer and fruits probably protect against gastric cancer. Most of the previous evidence was from case–control studies, which may be affected by recall and selection biases. More recently, several cohort studies have examined these associations. We conducted a systematic literature review of prospective studies on citrus fruits intake and risk of esophageal and gastric cancers.

**Methods:**

PubMed was searched for studies published until 1 March 2016. We calculated summary relative risks and 95 % confidence intervals (95 % CI) using random-effects models.

**Results:**

With each 100 g/day increase of citrus fruits intake, a marginally significant decreased risk of esophageal cancer was observed (summary RR 0.86, 95 % CI 0.74–1.00, 1,057 cases, six studies). The associations were similar for squamous cell carcinoma (RR 0.87, 95 % CI 0.69–1.08, three studies) and esophageal adenocarcinoma (RR 0.93, 95 % CI 0.78–1.11, three studies). For gastric cancer, the nonsignificant inverse association was observed for gastric cardia cancer (RR 0.75, 95 % CI 0.55–1.01, three studies), but not for gastric non-cardia cancer (RR 1.02, 95 % CI 0.90–1.16, four studies). Consistent summary inverse associations were observed when comparing the highest with lowest intake, with statistically significant associations for esophageal (RR 0.77, 95 % CI 0.64–0.91, seven studies) and gastric cardia cancers (RR 0.62, 95 % CI 0.39–0.99, three studies).

**Conclusions:**

Citrus fruits may decrease the risk of esophageal and gastric cardia cancers, but further studies are needed.

**Electronic supplementary material:**

The online version of this article (doi:10.1007/s10552-016-0755-0) contains supplementary material, which is available to authorized users.

## Introduction

Esophageal and gastric cancers are the eight and the fifth most common cancers worldwide, respectively [1]. Esophageal cancer accounted for 456,000 new cancer cases in 2012 [1]—it is the sixth most common cause of cancer mortality, with 400,000 deaths in 2012 reflecting its poor prognosis, and has a 5-year survival rate of 15–25 % [2]. Squamous cell carcinoma (SCC) is the predominant histological type of esophageal cancer worldwide but in USA, UK, Australia, and some Western European countries, and the incidence of esophageal adenocarcinomas now exceeds that of SCC [3, 4]. Gastric cancer is more common in low- and middle-income countries, and although incidence rates are declining in most parts of the world, almost one million new cases occurred worldwide in 2012 [1]. The incidence of cancers of the gastric cardia has remained stable or increased at least in Western countries. Gastric cancer is usually diagnosed at advanced stages. This makes the disease the third leading cause of cancer death globally, with an estimated 723,000 deaths in 2012 [1].

Tobacco use is a risk factor for esophageal and gastric cancers. Alcohol and tobacco use are the main risk factors for esophageal SCC [5]. Due to close anatomical proximity and similar etiology, esophageal adenocarcinomas and cancers of the gastric cardia have other risk factors in common, including obesity and gastro-esophageal reflux disease [5, 6]. Helicobacter Pylori infection is the major risk factor for non-cardia gastric cancer. Approximately, 80 % of non-cardia gastric cancers are attributable to *Helicobacter Pylori* infection. Despite the possibility of preventing non-cardia gastric cancer by treating *H. Pylori* infection, there are concerns with possible adverse consequences of the antibiotic treatment, such as development of antibiotic resistance and alterations of the intestinal microbiota [7]. There is no effective screening for early detection of these cancers.

Diet may also play a role on the development of esophageal and gastric cancers. In 2007, the World Cancer Research Fund/American Institute for Cancer Research (WCRF/AICR) Second Expert Report concluded that there was evidence that high total intake of salt probably increases the risk of gastric cancer, and that vegetables and fruits intake probably protects against esophageal and gastric cancers [8]. With respect to fruit intake, recent meta-analyses of cohort studies reported significant inverse associations with gastric cancer [9] and esophageal SCC [10] but not with adenocarcinomas of the esophagus [11].

Citrus fruits are rich in vitamin C, and foods containing vitamin C were judged probably to protect against esophageal cancer in the WCRF/AICR Second Expert Report [8]. Much of the previous evidence on citrus fruits was based on case–control studies. More recently, a publication from an integrated network of case–control studies [12], conducted in Italy and Switzerland, reported a significantly inverse association between citrus fruits intake and risk of esophageal cancer.

A recent meta-analysis of cohort studies reported nonsignificant inverse association between citrus fruits intake and the risk of gastric cancer for the comparison of the highest versus the lowest intakes [9]. However, there is no recent meta-analysis of cohort studies on citrus fruits intake and risk of esophageal cancer or subtypes of esophageal and gastric cancers. As part of the WCRF/AICR Continuous Update Project (CUP) [13], we conducted a systematic literature review and meta-analysis of cohort studies to investigate the association between citrus fruits intake and the risk of esophageal cancer, adenocarcinomas and squamous cell carcinomas, and total gastric, cardia, and non-cardia gastric cancers.

## Methods

### Search strategy

All cohort studies identified in the systematic literature review for the WCRF/AICR Second Expert Report [8] were indexed in PubMed. Therefore, we updated the search using the same search strategy in PubMed for studies published until 1st March 2016. Searches for esophageal and gastric cancers were carried out separately following protocols that can be accessed at http://www.wcrf.org/int/research-we-fund/continuous-update-project-cup. In addition, reference lists of relevant reviews identified in the search and of the studies included in the meta-analysis were screened for any further publications.

### Study selection

The following inclusion criteria were applied for studies included in this meta-analysis: (a) cohort, nested case–control or case-cohort design; (b) reported estimates of the relative risk (hazard ratio, odds ratio, or risk ratio) with confidence intervals (CI); (c) reported quantifiable measure of citrus fruits intake. If several publications using the same study population were identified, the one with the largest number of cases was selected.

### Data extraction

The following data were extracted from each study: the first author’s last name, publication year, country in which the study was conducted, study name, follow-up period, sample size, sex, age, number of cases, dietary assessment method (type, number of food items, validation), exposure, frequency or amount of intake, associated RR and corresponding 95 % CI, and adjustment variables. The search and data extraction for the systematic literature reviews of esophageal cancer and gastric cancer prior to January 2006 was conducted by the WCRF/AICR Second Expert Report teams at the Pennsylvania State University and the University of Leeds, respectively [8]. The search and data extraction from January 2006 to 1 March 2016 was conducted by the CUP team at Imperial College London. All extracted data are stored in the CUP database [13].

### Statistical analyses

We conducted dose–response meta-analyses and summarized the associations for the highest compared to the lowest citrus fruits intake reported in the studies using random-effects models [14].

When not provided in the publications, the linear dose–response trends were derived from the natural logs of the RRs and CIs across categories of citrus fruits intake, using the method by Greenland and Longnecker [15]. For this method, the distribution of person-years, cases, RRs, and CIs for at least three categories is required. When not available, person-years per quantile were estimated by dividing total person-years by the number of quantiles. Means or medians of intake were assigned to each category, and when a study reported only the range of intake per category, the midpoint was estimated. For open-ended uppermost or lowermost intake categories, we computed the midpoint by assigning the width to match the nearest category. When intake was reported per unit of energy intake [16, 17], we estimated the absolute intake per quantile using the mean energy intake of the whole study population provided in the paper. When intake was reported in times or servings per day or per week, we used a standard portion size of 80 g to convert frequency to grams (http://www.wcrf.org/sites/default/files/protocol_oesophageal_cancer.pdf). The dose–response was expressed for an increment of 100 g/day of citrus fruits. We used the multivariable adjusted RR from each study. The EPIC study [18, 19] reported calibrated relative risk estimates to account for possible diet measurement error, and we used these calibrated risk estimates for the linear dose–response meta-analysis.

We first estimated summary RR for all esophageal and gastric cancers, respectively. For these analyses, the RRs for men and women were combined using fixed-effect meta-analysis before pooling. When RRs were reported by cancer subtypes only, we estimated the combined RR of gastric cardia and non-cardia or esophageal adenocarcinoma and squamous cell carcinoma using Hamling’s method [20]. The meta-analyses were also conducted by sex and cancer type, for which we combined the RRs of esophageal adenocarcinoma and gastric cardia cancers using fixed-effect models. The extent of heterogeneity in the meta-analyses was assessed using Cochran Q test and I^2^ statistics, with low and high heterogeneity extent indicated by *I*^2^ values below 30 % or substantially higher than 50 % [21].

Subgroup analyses were conducted to assess possible sources of heterogeneity, as well as study quality. The predefined factors to explore were sex, outcome type, geographic location, duration of follow-up, number of cases, publication year, and adjustment for confounders including smoking, alcohol intake and adiposity (as measured by BMI), when the number of studies allowed it.

Publication bias was assessed with Egger’s test [22] and visually by using funnel plot. All analyses were conducted using Stata version 12 software (Stata Corp, College Station, TX).

## Results

**Fig. 1:**
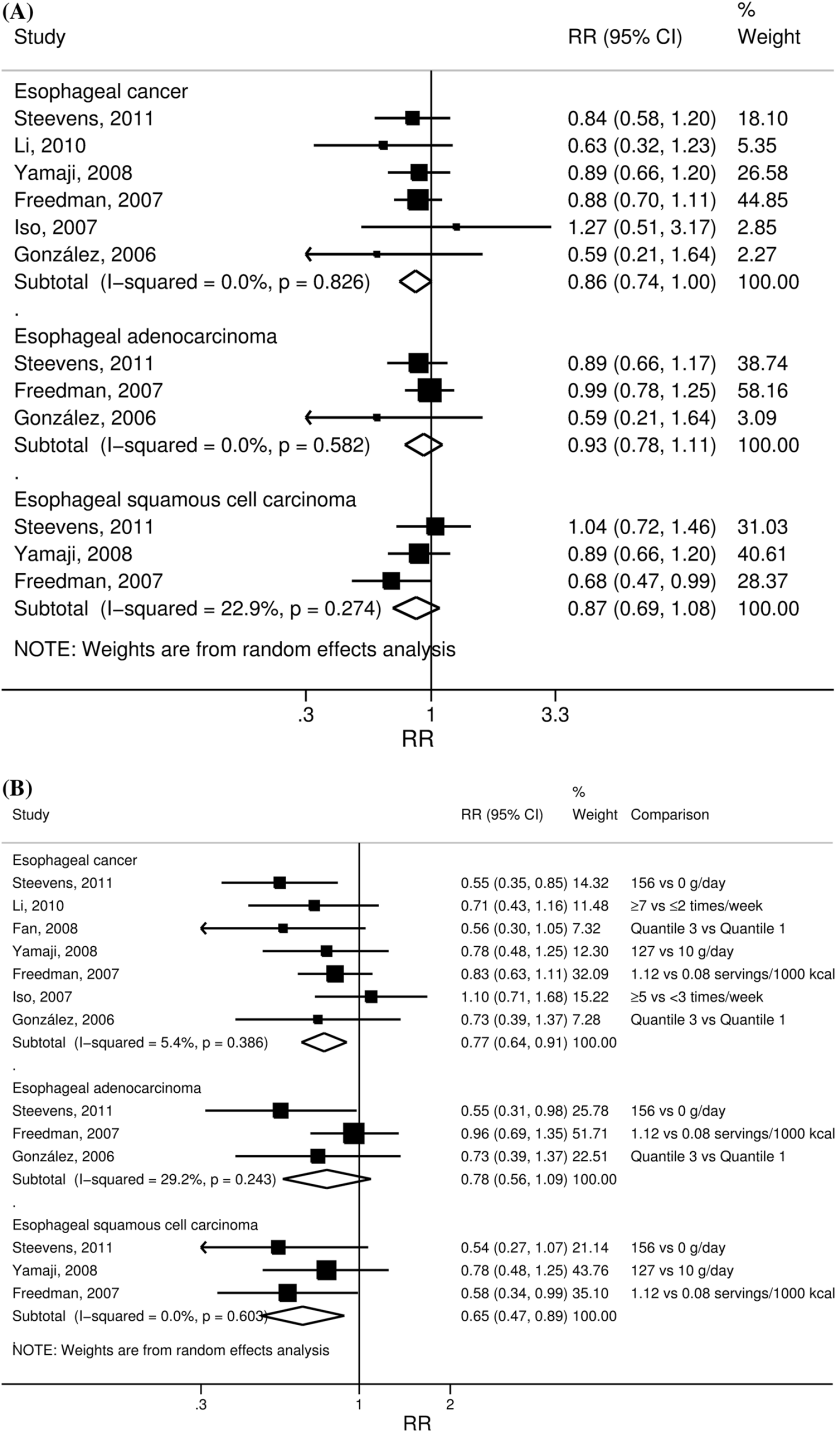
Summary RRs of esophageal cancer, esophageal adenocarcinoma, and squamous cell carcinoma per 100 g/day increase in citrus fruits intake (**a**) and in the highest versus lowest analysis (**b**)

**Fig. 2:**
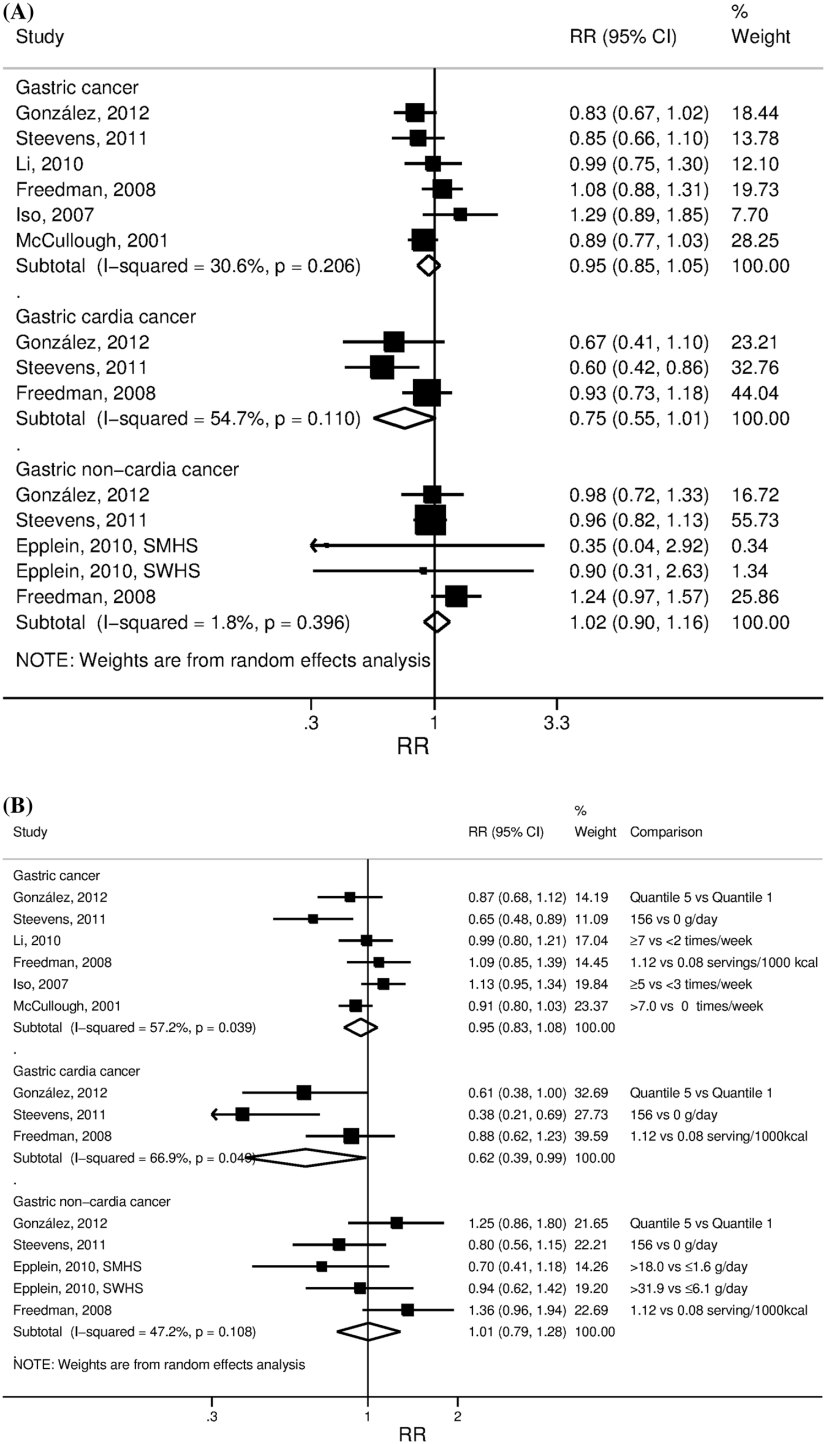
Summary RRs of gastric, gastric cardia and non-cardia cancers per 100 g/day increase in citrus fruits intake (**a**) and in the highest versus lowest analysis (**b**)

Flowcharts of the search are provided as an online resource (Fig. 1a, b). Seven potentially relevant cohort studies [16, 18, 23–27] on esophageal and eight studies (seven publications) [17, 19, 24–26, 28, 29] on gastric cancer were identified (Table 1). For the linear dose–response meta-analysis, one publication including two cohort studies [28] investigated non-cardia gastric cancer only and was excluded from the analysis of all gastric cancers; one study on esophageal cancer was also excluded because it did not provide quantifiable measure of exposure [23]. Hence, six studies [16, 18, 24–27] were included in the dose–response for esophageal cancer and six studies [17, 19, 24–26, 29] for gastric cancer (Figs. 1a, 2b).

**Table 1:** Prospective cohort studies of citrus fruits intake and esophageal and gastric cancer risk

Author, year, country (ref)	Study name, characteristics	Follow-up period (years of follow-up)	Study size, sex, number of cases	Dietary assessment	Outcome	Quantity	RR	Adjustment for confounders
*Esophageal cancer*								
Steevens 2011, Netherlands [26]	NLCS Case cohort Age: 55–69 years	1986–2002 (16.3)	4 035 Men and women, 144	Validated FFQ, 150 food items, fresh lemon juice, grapefruits, grapefruit juice, mandarins, oranges, fresh orange juice	Incidence Esophageal AC	156 versus 0 g/day Per 25 g/day	0.55 (0.31–0.98) Ptrend: 0.37 0.97 (0.90–1.04)	Age, sex, smoking status, cigarettes/day, smoking duration, alcohol, red meat, fish, vegetable, all other fruits
			101		Esophageal SCC	156 versus 0 g/day Per 25 g/day	0.54 (0.27–1.07) Ptrend: 0.38 1.01 (0.92–1.10)	
Li, 2010, Japan [25]	NHI Prospective Cohort Age: 40–79 years	1995–2003 (9)	42,470 Men and women, 151	Validated FFQ, 40 food items, citrus fruit	Incidence Esophageal cancer	≥7 versus ≤2 times/week	0.71 (0.43–1.16) Ptrend: 0.18	Age, sex, BMI, smoking, alcohol, employment, education, walking, exercise or sports, diabetes, gastric ulcer, hypertension, family history of cancer, energy intake, intake of tea, coffee, miso soup, rice, soybean, dairy products, fish, meat, vegetables, and other fruits
Yamaji, 2008, Japan [27]	JPHC Prospective Cohort Age: 40–69 years	1995/1998–2004 (7.7)	38 790 Men, 116	Validated FFQ, 138 food and beverage items, mandarin oranges, other oranges, 100 % orange juice	Incidence Esophageal SCC	127 versus 10 g/day Per 100 g/day	0.78 (0.48–1.25) 0.89 (0.66–1.20)	Age, study area, cigarette smoking, alcohol drinking
Freedman, 2007, USA [16]	NIH-AARP Prospective Cohort Age: 50 years	1995/1996–2000 (4.5)	490,802 Men and women, 213	Validated FFQ, 124 food items, oranges, tangerines, tangelos, grapefruits	Incidence Esophageal AC	1.12 versus 0.08 serving/ 1,000 kcal	0.96 (0.69–1.35)	Age, sex, BMI, alcohol, education, smoking dose, total energy intake, usual activity throughout the day, vigorous physical activity
			103		Esophageal SCC		0.58 (0.34–0. 99) Ptrend: 0.05	
Iso, 2007, Japan [24]	JACC Prospective cohort Age: 40–79 years	N/A-2003 (15)	43,011 Men, 139	Validated FFQ, 39 food items, citrus fruit	Mortality Esophageal cancer	≥5 versus < 3 times/week	1.18 (0.73–1.89)	Age, area of study
			59 504 Women, 25				0.80 (0.30–2.11)	
González, 2006, 10 European countries [18]	EPIC Prospective cohort Age: 35–70 years	1992/1998–2002 (6.5)	481 518 Men and women, 67	Country-specific validated questionnaires, 88–266 items; food record, citrus fruit, juices excluded	Incidence Esophageal AC	≥43.40 versus ≤10.68 g/day (M) ≥60.71 versus ≤17.43 g/day (W) Per 50 g/day	0.73 (0.39–1.37) Ptrend: 0.22 (observed) 0.77 (0.46–1.28) (calibrated)	Age, sex, center, education level, energy intake, height, leisure, physical activity, red meat intake, weight, work, physical activity, alcohol intake, processed meat intake, smoking
Author, year, country (ref)	Study name, characteristics	Follow-up period (years of follow-up)	Study size, sex, number of cases	Dietary assessment	Outcome	Quantity	RR	Adjustment for confounders
*Gastric cancer*								
González, 2012, 10 European countries [19]	EPIC Prospective Cohort Age: 35–70 years	1992/1998–2010 (11.02) 240 206 225	477 312 Men and women, 683	Country-specific validated questionnaires, 88–266 items; food record, citrus fruit, juices excluded	Incidence Gastric AC	103.6 versus 10.8 g/day (M) 84.2 versus 22.7 g/day (W) Per 50 g/day	0.87 (0.68–1.12) Ptrend: 0.07 0.91 (0.82–1.01) (calibrated) 0.97 (0.90–1.04) (observed)	Age, sex, BMI, center, educational level, energy intake, physical activity, total vegetable consumption, alcohol intake, other fruits, red and processed meat, smoking, other fresh fruits
			Never smokers Former smokers Current smokers			Highest versus lowest Per 50 g/day Highest versus lowest Per 50 g/day Highest versus lowest Per 50 g/day	(0.64–1.51) 1.00 (0.90–1.11) 0.90 (0.57–1.40) 1.01 (0.90–1.14) 0.69 (0.44–1.10) 0.86 (0.74–1.01)	
			201		Gastric cardia AC	103.6 versus 10.8 g/day (M) 84.2 versus 22.7 g/day (W) Per 50 g/day	0.61 (0.38-1.00) Ptrend: 0.01 0.82 (0.64–1.05) (calibrated) 0.85 (0.71–1.02) (observed)	
			323		Gastric non-cardia AC	103.6 versus 10.8 g/day (M) 84.2 versus 22.7 g/day (W) Per 50 g/day	1.25 (0.86–1.80) Ptrend: 0.46 0.99 (0.85–1.15) (calibrated) 1.03 (0.95–1.13) (observed)	
Steevens, 2011, Netherlands [26]	NLCS Case cohort Age: 55–69 years	1986–2002 (16.3)	4 035 Men and women, 156	Validated FFQ, 150 food items, fresh lemon juice, grapefruits, grapefruit juice, mandarins, oranges, fresh orange juice	Incidence Gastric cardia AC	156 versus 0 g/day Per 25 g/day	0.38 (0.21–0.69) Ptrend: 0.003 0.88 (0.81–0.97)	Age, sex, smoking status, cigarettes/day, smoking duration, alcohol, red meat, fish, vegetable, all other fruits
			460		Gastric non-cardia AC	156 versus 0 g/day Per 25 g/day	0.80 (0.56–1.15) Ptrend: 0.46 0.99 (0.95–1.03)	
Li, 2010, Japan [25]	NHI Prospective cohort Age: 40–79 years	1995–2003 (9)	42 470 Men and women, 806	Validated FFQ, 40 food items, citrus fruit	Incidence Gastric cancer	≥7 versus ≤2 times/week	0.99 (0.80–1.21) Ptrend: 0.90	Age, sex, BMI, smoking, alcohol, employment, education, walking, exercise or sports, diabetes, gastric ulcer, hypertension, family history of cancer, energy intake, intake of tea, coffee, miso soup, rice, soybean, dairy products, fish, meat, vegetables, and other fruits
Epplein, 2010, China [28]	SMHS Prospective cohort Age: 40–74 years	2002/2006–2007 (3.6)	59 247 Men, 132	Validated FFQ, 81 food items, tangerines, oranges, grapefruit	Incidence Distal (i.e., non-cardia) gastric cancer	>18.0 versus ≤1.6 g/day	0.70 (0.41–1.18) Ptrend: 0.34	Age, education level, smoking, total energy intake
	SWHS Prospective cohort Age: 40–70 years	1996/2000–2007 (9.2)	73 064 Women, 206	Validated FFQ, 77 food items, tangerines, oranges, grapefruit		>31.9 versus ≤ 6.1 g/day	0.94 (0.62–1.42) Ptrend: 0.86	
Freedman, 2008, USA [17]	NIH-AARP Prospective cohort Age: 50–71 years Retired	1995/1996–2000 (4.5)	490 802 Men and women, 198	Validated FFQ, 124 food items, oranges, tangerines, tangelos, grapefruits	Incidence Gastric cardia cancer	1.12 versus 0.08 serving/1,000 kcal	0.88 (0.62–1.23)	Age, sex, BMI, ethnicity, alcohol intake, cigarette dose, education, total energy, usual activity throughout the day, vigorous physical activity
			196		Gastric non-cardia cancer		1.36 (0.96–1.94)	
Iso, 2007, Japan [24]	JACC Prospective cohort Age: 40–79 years	N/A-2003 (15)	43 011 Men, 715	Validated FFQ, 39 food items, citrus fruit	Mortality Gastric cancer	≥5 versus <3 times/week	1.06 (0.86–1.30)	Age, area of study
			59 504 Women, 344				1.29 (0.95–1.74)	
McCullough, 2001, USA [29]	CPS II Prospective cohort Age: 30 years	1982–1996 (14)	436 654 Men, 910	FFQ, 32 food items, citrus fruit, juices	Mortality Gastric cancer	>7 versus 0–1.9 times/week	0.88 (0.75–1.03)	Age, BMI, educational level, family history of stomach cancer, multivitamin supplement, smoking habits, aspirin use, ethnicity/race, vitamin C supplement
			533 391 Women, 439			>7 versus 0–2.9 times/week	0.97 (0.78–1.21)	

Main characteristics of studies included in the linear dose–response meta-analysis

*AC* adenocarcinoma, *SCC* squamous cell carcinoma, *BMI* body mass index, *FFQ* Food Frequency Questionnaire, *NLCS* the Netherlands Cohort Study on diet and cancer, *NHI* Ohsaki National Health Insurance Cohort, *JPHC* Japan Public Health Center-based Prospective Study, *JAAC* Japan Collaborative Cohort study, *NIH-AARP* National Institute of Health (NIH)-AARP(formerly the American Association for Retired Persons) Diet and Health Study, *EPIC* European Prospective Investigation into Cancer and Nutrition, *SMHS* Shanghai Men’s Health Study, *SWHS* Shanghai Women’s Health Study

Main study characteristics are shown in Table 1. Citrus fruits intake was assessed using food frequency questionnaires. The definition of citrus fruits exposure varied slightly across the studies; in three studies, it included citrus fruits juice [26, 27, 29] (Table 1).

All measures of association included in the meta-analyses were adjusted for multiple confounding factors, albeit defined differently in the studies, including alcohol [16–19, 25–27], BMI and physical activity [16–19, 25, 29], socioeconomic status [16, 18, 19, 25, 29], smoking status [16–19, 25–27, 29], number of cigarettes [11, 16–19, 26, 27], and duration of smoking [19, 26, 27] with the exception of a Japanese study with cancer mortality as endpoint that adjusted only for age and geographic area [24]. None of the studies adjusted for gastric reflux disease (GERD), Table 3. The lack of information about gastric reflux was indicated in one publication [16]. One study on gastric cancer mortality [29] investigated regular use of antacids but did not include it in the final model due to lack of confounding.

Gastric or esophageal cancers were primary outcomes in all but two studies [24, 25] that reported on multiple cancer sites.

Five studies were conducted in Asia [24, 25, 27, 28], two in Europe [18, 19, 26], and two in North America [16, 17, 29] (Table 1). All studies were included men and women apart from Yamaji et al., 2008 [27] which was only included men (Table 1). A summary of the results of meta-analyses by cancer type is presented in Table 2.

**Table 2:** Summary table of meta-analyses of citrus fruits and esophageal and gastric cancers

Cancer type	Esophageal cancer	Esophageal squamous cell carcinoma	Esophageal adenocarcinoma	Esophageal adenocarcinoma, gastric cardia	Gastric cancer	Gastric cardia cancer	Gastric non-cardia cancer
*Linear dose–response meta-analysis per 100* *g/day*							
No. of studies	6	3	3	3	6	3	4
No. of cases	1,057	320	422	1,348	4,907	555	1,317
Person-years	7,513,150	2,542,187	5,354,570	7,507,530	22,949,089	7,507,530	8,393,008
RR (95 % CI)	0.86 (0.74–1.00)	0.87 (0.69–1.08)	0.93 (0.78–1.11)	0.83 (0.67–1.02)	0.95 (0.85–1.05)	0.75 (0.55–1.01)	1.02 (0.90–1.16)
*I* ^2^, *P*_heterogeneity_	0 %, 0.83	23 %, 0.27	0 %, 0.58	50 %, 0.14	31 %, 0.21	55 %, 0.11	2 %, 0.40
*Highest versus lowest analysis*							
No. of studies	7	3	3	3	6	3	4
No. of cases	1 158	320	422	1 348	4 907	555	1 317
RR (95 % CI)	0.77 (0.64–0.91)	0.65 (0.47–0.89)	0.78 (0.56–1.09)	0.67 (0.44–1.01)	0.95 (0.83–1.08)	0.62 (0.39–0.99)	1.01 (0.79–1.28)
*I* ^2^, *P*_heterogeneity_	5 %, 0.39	0 %, 0.60	29 %, 0.24	77 %, 0.01	57 %, 0.04	67 %, 0.05	47 %, 0.11

### Esophageal cancer

Six studies [16, 18, 24–27] with a total of 1,057 cases among 1,160,130 participants were included in the linear dose–response meta-analysis. Citrus fruit was inversely associated with esophageal cancer risk; the association was statistically significant only in the highest versus lowest analysis. The summary RR for an increase of 100 g/day of citrus fruits intake was 0.86 (95 % CI 0.74–1.00), with no evidence of heterogeneity (*I*^2^ = 0 %, *P*_heterogeneity_ = 0.83) (Fig. 1a). There was no evidence of publication or small study bias (*p* = 0.55). The summary RR for the highest compared with the lowest intake was 0.77 (95 % CI 0.64–0.91), with low heterogeneity (*I*^2^ = 5 %, *P*_heterogeneity_ = 0.39) (Fig. 1b).

In analyses by cancer type, three studies could be included in the analyses of adenocarcinoma [16, 18, 26] and SCC [16, 26, 27] of the esophagus, respectively. Similar not statistically significant inverse associations were observed for both cancer types in linear dose–response meta-analyses. The summary RR per 100 g/day increase in citrus fruits intake was 0.93 (95 % CI 0.78–1.11, 422 cases, three studies) for esophageal adenocarcinoma, with no evidence of heterogeneity (*I*^2^ = 0 %, *P*_heterogeneity_ = 0.58) and 0.87 (95 % CI 0.69–1.08, 320 cases, three studies) for SCC with low heterogeneity (*I*^2^ = 23 %, *P*_heterogeneity_ = 0.27) (Fig. 1a). The summary RR for the highest compared with the lowest intake was 0.78 (95 % CI 0.56–1.09) for adenocarcinomas and 0.65 (95 % CI 0.47–0.89) for SCC (Fig. 1b).

Only two studies in men [24, 27], one incidence and one on mortality from esophageal cancer and one study on esophageal cancer mortality in women [24] were available. There is not enough data to examine the association of citrus fruits and esophageal cancer risk by sex (Table 3).

**Table 3:** Subgroup meta-analyses of citrus fruits and risk of esophageal and gastric cancers

Per 100 g/day	Esophageal cancer				Gastric cancer			
	*N*	RR (95 % CI)	*I* ^2^ (%)	*P*_heterogeneity_	*N*	RR (95 % CI)	*I* ^2^ (%)	*P*_heterogeneity_
All studies	6	0.86 (0.74–1.00)	0	0.83	6	0.95 (0.85–1.05)	31	0.21
Sex								
Men	2	0.93 (0.70–1.24)	0	0.34	2	0.91 (0.76–1.09)	8	0.30
Women	1	0.63 (0.08–5.23)	–	–	2	1.20 (0.67–2.15)	65	0.09
Outcome type								
Incidence	5	0.85 (0.73–0.99)	0	0.84	4	0.93 (0.82–1.07)	23	0.27
Mortality	1	1.27 (0.51–3.17)	–	–	2	1.03 (0.73–1.46)	70	0.07
Geographic location								
Asia	3	0.87 (0.67–1.13)	0	0.45	2	1.10 (0.85–1.41)	21	0.26
Europe	2	0.80 (0.57–1.13)	0	0.54	2	0.84 (0.71–0.98)	0	0.86
North America	1	0.88 (0.70–1.11)	–	–	2	0.97 (0.81–1.16)	55	0.13
Europe and North America	3	0.86 (0.71–1.04)	0	0.75	4	0.91(0.82–1.02)	23	0.28
Duration of follow-up								
<10 years	4	0.85 (0.72–1.02)	0	0.70	2	1.05 (0.89–1.23)	0	0.62
≥10 years	2	0.88 (0.63–1.24)	0	0.40	4	0.90 (0.79–1.03)	32	0.22
Number of cases								
<100	1	0.59 (0.21–1.65)	–	–		–		
100–<200	3	0.87 (0.67–1.13)	0	0.45		–		
200–<500	2	0.87 (0.71–1.05)	0	0.81	1	1.08 (0.88–1.31)	–	–
500–<1,000		–			3	0.87 (0.76–1.00)	0	0.60
≥1,000		–			2	1.03 (0.73–1.46)	70	0.07
Publication year								
<2,010	4	0.89 (0.74–1.06)	0	0.75	3	1.02 (0.85–1.24)	56	0.10
≥2,010	2	0.78 (0.57–1.08)	0	0.47	3	0.87 (0.76–1.00)	0	0.60
*Adjustment for confounders*								
Socioeconomic status								
Yes	3	0.84 (0.68–1.04)	0	0.52	4	0.93 (0.83–1.04)	21	0.29
No	3	0.89 (0.71–1.11)	0	0.70	2	1.02 (0.69–1.53)	69	0.07
Smoking								
Yes	5	0.85 (0.73–0.99)	0	0.84	5	0.92 0.84–1.01	4	0.39
No	1^a^	1.27 (0.51–3.17)	–	–	1^a^	1.29 0.89–1.85	–	–
Alcohol intake								
Yes	5	0.85 (0.73–0.99)	0	0.84	4	0.93 (0.82–1.07)	23	0.27
No	1^a^	1.27 (0.51–3.17)	–	–	2^b^	1.03 (0.73–1.46)	70	0.07
BMI								
Yes	3	0.84 (0.68–1.04)	0	0.52	4	0.93 (0.83–1.04)	21	0.29
No	3	0.89 (0.71–1.11)	0	0.70	2	1.02 (0.69–1.53)	69	0.07
Physical activity								
Yes	3	0.84 (0.68–1.04)	0	0.52	3	0.96 (0.81–1.13)	39	0.20
No	3	0.89 (0.71–1.11)	0	0.70	3	0.94 (0.78–1.14)	46	0.16
Total energy intake								
Yes	3	0.84 (0.68–1.04)	0	0.52	3	0.96 (0.81–1.13)	39	0.20
No	3	0.89 (0.71–1.11)	0	0.70	3	0.94 (0.78–1.14)	46	0.16
Ethnicity								
Yes		–			2	0.97 (0.81–1.16)	55	0.13
No	6	0.86 (0.74–1.00)	0	0.83	4	0.93 (0.79–1.11)	37	0.19

^a^Minimally adjusted study for age and study area [24]

^b^Minimally adjusted study for age and study area [24] and another study which did not include alcohol intake in the final model but tested that it did not confound the association [29]

In subgroup analysis (all esophageal cancers), no differences emerged across study characteristics, including adjustment factors (Table 3). There is some suggestion that more adjusted studies tend to report stronger associations, but the number of studies is low. A positive not significant association was observed in the only study [24] that did not adjust for tobacco, smoking, and alcohol intake in which the outcome was mortality for esophageal cancer. When this study was omitted from the analysis, the summary RR for an increase of 100 g/day of citrus fruits intake was 0.85 (95 % CI 0.73–0.99) with no heterogeneity.

### Gastric cancer

Six studies [17, 19, 24–26, 29] investigated the association between citrus fruits intake and gastric cancer risk with a total of 4,907 cases among 2,087,179 participants. No significant association with gastric cancer was observed. The summary RR per 100 g/day increment was 0.95 (95 % CI 0.85–1.05), with moderate [21] heterogeneity (*I*^2^ = 31 %, *P*_heterogeneity_ = 0.34) (Fig. 2a). The summary RR for the highest compared to the lowest intake was 0.95 (95 % CI 0.83–1.08) with evidence of heterogeneity (*I*^2^ = 57 %, *P*_heterogeneity_ = 0.04) (Fig. 2b).

In subgroup analyses by cancer type, inverse association was observed for cancers of the gastric cardia, but not for non-cardia gastric cancers. Three studies [17, 19, 26] investigated the association between citrus fruits intake and gastric cardia cancer risk with a total of 555 cases among 972,149 participants. The summary RR for 100 g/day increment was 0.75 (95 % CI 0.55–1.01), with moderate [21] heterogeneity (*I*^2^ = 55 %, *P*_heterogeneity_ = 0.11) (Fig. 2a), and it was 0.62 (95 % CI 0.39–0.99) comparing the highest with lowest intake, with high heterogeneity (*I*^2^ = 67 %, *P*_heterogeneity_ = 0.05) (Fig. 2b).

Five studies [17, 19, 26, 28] investigated the association between citrus fruits intake and non-cardia gastric cancer risk with a total of 1,317 cases among 1,104,460 participants. The summary RR for 100 g/day increment was 1.02 (95 % CI 0.90–1.16), with low heterogeneity (*I*^2^ = 2 %, *P*_heterogeneity_ = 0.4) (Fig. 2a), and it was 1.01 (95 % CI 0.79–1.28) for the highest compared with the lowest intake (Fig. 2b).

When the analyses were restricted to the three studies [17, 19, 26] that reported on both cardia and non-cardia gastric cancers, the RRs for an increase of 100 g/day were 0.75 (95 % CI 0.55–1.01) and 1.04 (95 % CI 0.89–1.22), respectively.

It was not possible to formally explore the source of heterogeneity in the analyses on cardia gastric cancer. Visual inspection of the forest plot shows that heterogeneity is driven by the American NIH-AARP study [17] that reported no association of citrus fruits with cardia gastric cancer. The reasons for the different results are unclear. The NIH-AARP study [17] categorized intake by servings/1,000 kcal, whereas the two other studies [19, 26] reported in continuous increments in g/day.

### Esophageal adenocarcinoma and gastric cardia cancers

We estimated the summary RR of esophageal adenocarcinomas and gastric cardia cancers (three studies, five publications) [16–19, 26]. When combined, these cancers totaled to 1,348 cases among 5,268,049 participants. The summary RR per 100 g/day increment was 0.83 (95 % CI 0.67–1.02), with moderate [21] heterogeneity (*I*^2^ = 50 %, *P*_heterogeneity_ = 0.14) (Table 2). The summary RR was 0.67 (95 % CI 0.44–1.01) for the highest compared with the lowest intake (Table 2).

Summary risk estimates observed in subgroup analyses for all gastric cancers were mostly similar to that in the overall analysis, with exceptions in some subgroups where a positive association was observed. Estimates of risk were below 1 in studies adjusted for smoking and alcohol and BMI but not in the unadjusted studies (Table 3). Significant associations were observed in subgroup analyses for all gastric cancers among European studies [19, 26] and studies with 500–<1,000 cases [19, 25, 26]. Inverse not significant associations were observed in men but not in women (2 studies) [24, 29]. There was no evidence of small study effects such as publication bias (*p* = 0.25).

### Interaction with smoking

One study reported on the interaction of smoking status and citrus fruits intake in relation to esophageal or gastric cancers. In the EPIC study [19], the inverse association of citrus fruits for gastric cancer was restricted to current smokers and not observed in never or former smokers (*p* for interaction =0.07). Other studies in the review explored the interaction of smoking and intake of total fruits and vegetables, or fruits. In general, no significant interactions with smoking were observed. In the NIH-AARP study, the risk estimates of adenocarcinoma and SCC for total fruits and vegetable intakes appeared similar in smokers, non-smokers, and current smokers [17]. In the study in Japanese men, esophageal SCC risk was inversely associated with total fruits or vegetables intake in never, current and former smokers [27]. In the Netherlands Cohort Study, slightly greater inverse associations of fruit intake with SCC and adenocarcinomas of esophagus and gastric cardia cancer were reported in smokers that in never smokers, but the interaction was not significant (*p* for interaction = 0.25; 0.15; and 0.49, respectively) [26]. In a Chinese study in men and women, a significant reduction in risk of distal gastric cancer from increased fruit intake was significant among ever smokers and inverse but nonsignificant in never smokers, but the interaction by smoking status was not statistically significant (*p* for interaction = 0.27) [28].

## Discussion

In these meta-analyses of cohort studies, citrus fruits intake was marginally associated with reduced risks of esophageal and gastric cardia cancers. No association with non-cardia gastric cancers was observed. Similar results were observed for adenocarcinomas and SCC of esophagus.

Citrus fruits are rich in vitamin C that could influence cancer risk by scavenging reactive oxygen species, protecting mucosal tissues from the damaging effects of oxidative stress, and inhibiting nitrosamine formation in the stomach [30]. The results of this meta-analysis are consistent with the inverse association of prediagnostic plasma vitamin C concentration and risk of gastric cardia cancer (215 cases) observed in the EPIC study [31] and in a study in a high-risk Chinese population (467 cases) [32]. In the EPIC study, the associations were more pronounced for gastric cardia than non-cardia cancer, although the associations were not statistically significant when stratified by subtype. Further evidence is provided by the Shandong Intervention Trial of vitamin supplementation (vitamin C, E and selenium), in which supplemented individuals had a lower risk of esophageal and gastric cancers [33] and in a meta-analysis of 20 randomized controlled trials of antioxidant supplementation (vitamins A, C, E, and selenium) inverse but not significant lower risk of gastrointestinal cancers was observed [34]. In the NIH-AARP, use of vitamin C supplements was associated with reduced risk of gastric non-cardia adenocarcinomas, but no association was observed with multivitamin supplements use that usually contains vitamin C [35]. Finally, in recent meta-analyses, total fruit intake was associated with significantly lower risk of gastric cancer [9] and esophageal squamous cell carcinoma [10].

In addition to high vitamin C content, citrus fruits contain a wide range of bioactive compounds such as citrus flavonoids, carotenoids, and limonoids. Experimental studies have demonstrated that these bioactive components may protect DNA, regulate cell growth, and induce apoptosis [36–38].

The main limitation of this meta-analysis is the small number and limited power of published studies on citrus fruits intake, esophageal and gastric cancer risks, and the unexplained heterogeneity of the inverse association of citrus fruits intake and gastric cancer cardia in the three studies identified [17, 19, 26].

The observed inverse associations could be due to residual confounding by smoking. In the EPIC study [19], the inverse association of citrus fruits for gastric cancer was restricted to current smokers and not observed in never or former smokers (*p* for interaction =0.07). However, other studies included in the meta-analysis [16, 17, 19, 26] reported no evidence of interaction of effect modification by smoking status. In the NIH-AARP, the association between fruit and vegetable intake with ESCC [16] was similar in the limited number of non-drinkers and non-smokers; in a study on gastric cancer [17], there was no evidence of effect modification by cigarette smoking status or alcohol drinking; in the NLCS study, the risk estimates for total fruit intake and risk of all types of gastric and esophageal cancers were further below 1 in current smokers compared to never and former smokers, but the interaction was not significant (*p* for interaction >0.15) [26]. On the other hand, smokers tend to eat less fruits and vegetables [39, 40], have lower concentration of serum antioxidants [41], and may benefit more from higher citrus fruits intake [27].

Measurement error of diet may have attenuated the risk estimates. Only the EPIC cohort corrected for dietary measurement error [18, 19]. When non-calibrated risk estimates from the EPIC cohort were used in the sensitivity analysis, the association became significant for gastric cardia cancer (RR 0.75; 95 % CI 0.57–0.99), and the risk estimates did not change for esophageal cancer and remained similar for all gastric cancers (RR 0.96; 95 % CI 0.88–1.05) and gastric non-cardia cancer (RR 1.04; 95 % CI 0.94–1.16). Strengths of this meta-analysis include the prospective design of the included studies, which are less prone to bias than other observational studies, detailed dose–response and categorical meta-analyses, and the increased statistical power to detect modest but statistically significant inverse associations.

## Conclusions

In conclusion, there is evidence from cohort studies that citrus fruits may decrease the risk of esophageal and cardia gastric cancers, but the data are still limited.

## Electronic supplementary material

Below is the link to the electronic supplementary material.

Supplementary material 1 (DOCX 40 kb)
