# Supplementary material for: An update of the WCRF/AICR systematic literature review on esophageal and gastric cancers and citrus fruits intake
Source: Cancer Causes Control. 2016 May 6;27:837–51. doi: 10.1007/s10552-016-0755-0 (PMC4923099; doi:10.1007/s10552-016-0755-0)
Supplement: Supplementary file 1 — Supplementary material 1 (DOCX 40 kb) [file 10552_2016_755_MOESM1_ESM.docx]

*Article title:*

An update of the WCRF/AICR systematic literature review on oesophageal and gastric cancers, and citrus fruits intake

*Journal name:* Cancer Causes & Control

*Authors:*

Snieguole Vingeliene, Doris S. M. Chan, Dagfinn Aune, Ana R. Vieira, Elli Polemiti¹, Christophe Stevens, Leila Abar, Deborah Navarro Rosenblatt, Darren C. Greenwood and Teresa Norat

*Corresponding author:*

Mrs Snieguole Vingeliene, Department of Epidemiology and Biostatistics, School of Public Health, Faculty of Medicine, Imperial College London, St. Mary's Campus, Norfolk Place, London, W2 1PG, UK.

E-mail: [s.vingeliene@imperial.ac.uk](mailto:s.vingeliene@imperial.ac.uk)

**Fig. 1a** and **b**. Flow charts of the search for oesophageal (a) and stomach cancer publications (b) from January 1^st^ 2006 to March 1^st^ 2016

**Fig. 1a**

347 publications excluded:

139 with no association of interest

57 reviews/no original data

77 meta-analyses

8 letter/editorial/comments

1 no measure of the association

5 pooled analyses

1 ecological study

59 case-control studies

303492 publications excluded on the basis of title and abstract

354 publications retrieved and assessed for inclusion

7^a^ publications reported on association between citrus fruit intake and risk of gastric cancer and were included in the meta-analysis:

6 prospective cohort studies

1 case-cohort study

375 publications excluded:

105 with no association of interest

39 reviews/no original data

64 meta-analyses

5 letter/editorial/comments

2 no measure of the association

12 pooled analyses

7 ecological studies

1 cross-sectional study

140 case-control studies

8127 publications excluded on the basis of title and abstract

382 publications retrieved and assessed for inclusion

7 publications reported on association between citrus fruit intake and risk of oesophageal cancer

8509 potentially relevant publications identified

6^a^ publications included in the meta-analysis:

5 prospective cohort studies

1 case-cohort study

**Fig. 1b**

303846 potentially relevant publications identified

^a^ Includes one publication on gastric cancer and none on oesophageal cancer from the WCRF/AICR Second Expert Report, others were superseded.
